# Kcnv2 E151X Mouse Captures Hallmarks of KCNV2‐Associated Retinal Dystrophy

**DOI:** 10.1111/ceo.70163

**Published:** 2026-09-01

**Authors:** Nermina Xhaferri, Sumit Biswas, Benjamin Davies, Moritz Lindner

**Affiliations:** ^1^ Institute of Physiology and Pathophysiology, Department of Neurophysiology Philipps‐University Marburg Marburg Germany; ^2^ Francis Crick Institute London UK; ^3^ Department of Ophthalmology University Hospitals of Giessen and Marburg Marburg Germany

**Keywords:** electroretinography, inherited retinal dystrophy, KCNV2, KCNV2‐associated retinopathy, photoreceptors

## Abstract

**Background:**

KCNV2‐associated retinopathy is a rare inherited retinal dystrophy caused by variants in the KCNV2 gene, leading to disrupted photoreceptor behaviour and progressive deterioration of vision. Patients have characteristic electroretinography abnormalities, including reduced cone response, delayed and reduced rod response to low light flashes and paradoxically large rod‐driven response to bright flashes of light. To model this condition, we have generated a Kcnv2 E151X mouse line and assessed its structural and functional retinal features.

**Methods:**

We have employed CRISPR/Cas 9 gene editing technology to generate a mouse line with an early stop mutation in position E151—orthologous to the commonly encountered E143X mutation in humans—and performed a combination of immunohistochemistry and Western blot to confirm the absence of the full‐length KCNV2—encoded protein, Kv8.2. Next, to assess how closely it models the human disease, we have characterised the KCNV2 mutant mouse line at histological and functional levels, via immunohistochemistry and electroretinography experiments, respectively.

**Results:**

Kcnv2 mutant mice showed markedly reduced photopic responses and reproduced the supernormal rod phenotype described in affected individuals. In the morphological context, mutant retinas demonstrated strong glial fibrillary acidic protein upregulation together with reduced cone arrestin positive cell counts and photoreceptor layers, indicating photoreceptor loss.

**Conclusions:**

The Kcnv2 mutant mouse line replicates key functional and structural hallmarks of KCNV2‐associated retinopathy. This model provides a relevant platform for mechanistic studies and preclinical evaluation of gene‐based or pharmacological therapies targeting cone and rod photoreceptor dysfunction.

## 1 Introduction

KCNV2‐associated retinopathy, previously known as cone dystrophy with supernormal rod response (CDSRR), is an inherited retinal disorder characterised by a slowly progressive deterioration of visual function. It was first described in 1983 by Gouras et al. [1] and subsequently linked to mutations in the KCNV2 gene [2, 3]. This encodes for a voltage‐gated potassium channel subunit, K_v_8.2, which is expressed in rod and cone photoreceptors. While KCNV2‐associated retinopathy is overall rare, it is one of the commonest causes of autosomal recessive cone dystrophies [4] and the slow rate of disease progression leaves ample room for therapeutic intervention [5].

A recent study on mouse and primate photoreceptors [6] has shown that K_v_8.2 interacts with K_v_2.1 in rod inner segments and with both K_v_2.1 and K_v_2.2 in cone inner segments, giving rise to I_K,x_, the conductance that physiologically counterbalances the constant influx of positive charge through CNG channels (‘dark‐current’) in resting photoreceptors. K_v_2.1 homomers and K_v_8.2/K_v_2.1 heteromers differ in their voltage‐dependence of activation and inactivation. Heteromeric K_v_8.2/K_v_2.1 channels typically activate and inactivate at more hyperpolarised potentials than K_v_2.1 homomers. Moreover, the total conductance of K_v_8.2/K_v_2.1 channels is lower than that of K_v_2.1 homomers and the activation time is shortened [2, 7, 8]. The change in electrophysiological properties in the absence of K_v_8.2 translates to distinct features in the electroretinograms of the patients including the diagnostic supranormal scotopic b‐wave response to bright flash. Other typical electroretinographic observations include delayed and decreased scotopic responses to low‐flash intensities and markedly reduced to absent photopic responses. Another characteristic finding is the broadening of the a‐wave trough following scotopic bright‐flash stimulation [3, 9, 10, 11, 12].

Different reports collectively highlight the diversity of pathogenic Kcnv2 mutations including a variety of nonsense (E73X, Q76X, E143X, E148X, K260X, Q287X), missense (E184K, E184V, G461R), frameshift (K120fsX371) and deletions (D339_V341del, gross deletions) [3, 10, 13, 14]. Thereof, E143X is the commonest single pathogenic mutation [15] leading to an early truncation of K_v_8.2 inside the tetramerisation domain [16].

Patients with KCNV2 retinopathy typically present in the first two decades of their life with decreased visual acuity, colour vision abnormalities, photophobia, and night blindness. As shown by fundoscopy and clinical imaging, structural alterations in cones are observed early in the course of the disease while the rod‐dominated peripheral retina appears generally normal [3, 17].

Despite the growing insights into the genetic underpinnings of KCNV2 retinopathy, the precise molecular mechanisms by which KCNV2 mutations lead to retinal dysfunction and ultimately cone death remain poorly understood. Critical questions still remain unanswered: How do KCNV2 mutations affect I_k,x_? What are the molecular pathways driving retinal cell death and why are cones predominantly affected? Could a medical or gene‐therapeutic intervention aiming to restore physiological I_K,x_ only alleviate symptoms or also prevent cone loss?

In this study, we have generated and characterised a novel mouse model carrying the E151X mutation, orthologous to E143X in humans, not only to address what happens at the basis of the disease with a model that better resembles human disease cases than previous models, but also to evaluate its potential utility as a platform for developing novel therapeutic approaches. Via a combination of immunohistochemistry, Western Blot and electroretinogram experiments, we have shown that these mice closely mimic the phenotype of KCNV2‐associated retinopathy patients and provide a good model to elucidate the pathophysiological mechanisms of this blinding disease and pave the way for gene therapy strategies.

## 2 Methods

### 2.1 Animal Procedures

All procedures were performed with the approval of the Giessen Regional Council Animal Health Authority (File No: G19/2023) and the UK Home Office (PAA2AAE49), respectively, and in accordance with the ARVO Statement for the Use of Animals in Ophthalmic and Vision Research. Wild‐type C57BL/6J mice and Kcnv2 mice (Kcnv2 WT, Kcnv2^E151X/E151X^, Kcnv2^WT/E151X^) aged from 1 month to 1 year old, both males and females, were used in this study. Wild‐type C57BL/6J mice were obtained from Charles River Laboratories (Sulzfeld, Germany) and Kcnv2 mice were an in‐house breeding colony. Animals were housed in a temperature‐controlled environment with a 12‐h light/dark cycle with no restriction on food and water. Mice were back‐crossed for at least four generations before being used in experiments.

### 2.2 Generation of Kcnv2E151X Mice

Generation of the mouse line carrying the E143X mutation in the *Kcnv2* (NM_183179.1) gene (*Kcnv2* ^*em1Mlin*^, *Kcnv2* ^*E151X*^ hereafter) was performed following established procedures [18] utilising CRISPR/Cas9 gene editing technology. In brief, a target site (5′‐GTGCCTCGTAGTCATCACAC‐3′) for the nuclease on exon 1 (ENSMUSE00000395161) of the *Kcnv2* gene, overlapping the E151 residue, was identified using the CRISPOR design algorithm (http://crispor.tefor.net/). A single guide RNA (sgRNA) was synthesised by Synthego (Redwood City, CA, USA) and assembled into a ribonucleoprotein complex with Cas9 nuclease. A 139 nt single‐stranded oligodeoxynucleotide (ssODN) designed to introduce the E151X mutation (5′‐AAACGCGCCTGGGTCGCCTGGCCACCTCCACCACTCGCAGAGGCCAGCTGGGtCTGTGTGATGACTACtAGGCACAGACAGACGAGTACTTCTTTGACCGTGACCCAGCGGTCTTCCAGCTCATCTACAACTTCTACAC‐3′) was used as the template for homology‐directed repair. The CRISPR/Cas9 RNP and ssODN were introduced into fertilised C57BL/6J zygotes via electroporation as previously described [18] and cultured overnight. The resulting two‐cell stage embryos were surgically implanted into foster females. From the offspring, two founder mice which had successfully incorporated the E151X mutation were identified and subsequently bred with wild‐type C57BL/6J mice to produce heterozygous F1 mice. The transmission of the E151X mutation was verified through Sanger sequencing.

### 2.3 Genotyping

Mouse genomic DNA was extracted from ear notches or tail tips by incubation in 50 mM NaOH for 1–1.5 h at 95°C. Subsequently, a 477 bp region within exon 1 of *Kcnv2*, encompassing the target codon, was amplified via PCR (DreamTaq PCR Master Mix, Thermo Fisher, Waltham, MA, USA), utilising primers Kcnv2‐F (5′‐CTC ACA AGC CTC CTG GAT GG‐3′) and Kcnv2‐R (5′‐TCC ATA GAA GCG CAT GTC CC‐3′). PCR products were digested with BfaI (New England Biolabs, Cat. No. R0568S, Ipswich, MA, USA), yielding two fragments of 294 and 183 bp for the E151X‐allele and one 477 bp fragment for the wild‐type allele.

### 2.4 Immunohistochemistry

For immunohistochemistry experiments, tissue was collected at different timepoints spanning from 4 to 52 weeks of age. Following dark adaptation for at least 20 min, mice were anaesthetised using 5% isoflurane in 0.2 L/min oxygen and then sacrificed by decapitation. Two different tissue fixation strategies were used. The long fixation protocol has been described previously [19]. In brief, eyes were carefully removed and fixed in 4% paraformaldehyde (PFA, Thermo Fischer Scientific, Cat. No. 28906, Waltham, MA, USA) in phosphate‐buffered saline (PBS) for 24 h and transferred to a 30% sucrose solution. The eyes were then embedded in an optimal cutting temperature (OCT) compound (Sakura, Finetek, Torrance, CA, USA) and cryopreserved using either dry ice or isopropanol at −40°C. The short fixation protocol involved removing the eyes and carefully extracting the retinas in ice‐cold PBS. The extracted retinas were fixed in 4% PFA in PBS for 30 min and then placed in a 30% sucrose solution until fully immersed. Subsequently, the retinas were transferred to a mixture of 30% sucrose and OCT in a 3:1 ratio before being embedded in undiluted OCT compound and cryopreserved as above. For both protocols, the OCT‐embedded retinal sections were cut at a thickness of 20–25 μm, using a Leica CM1850 cryostat and stored at −20°C until further use. Immunofluorescence staining was performed as previously described [20] using the antibodies and dilutions shown in Table 1. Nuclei were counterstained with DAPI for 10 min. Finally, sections were mounted using Prolong Gold antifade mounting medium and coverslipped for imaging.

**TABLE 1:** List of primary and secondary antibodies used in this study.

Target	Host	Source	Dilution
Primary			
Kcnv2^a^	Mouse	Antibodies Incorporated, Cat. No. 75‐435, RRID: AB_2651161	1:400
Kcnv2	Rabbit	Sigma‐Aldrich, Cat. No. HPA031131, RRID: AB_10609996	1:400
GFAP	Rabbit	Abcam, Cat. No. ab7260, RRID: AB_305808	1:1000
Cone arrestin	Rabbit	Millipore, Cat. No. AB15282, RRID: AB_1163387	1:500
Secondary			
Mouse	Donkey	Invitrogen Alexa Fluor 488, Cat. No. A‐21202, RRID: AB_141607	1:1000
Rabbit	Donkey	Invitrogen Alexa Fluor 488, Cat. No. A‐21206, RRID: AB_2535792	1:1000
Mouse	Donkey	Invitrogen Alexa Fluor 568, Cat. No. A‐10037, RRID: AB_11180865	1:1000

^a^ Full amino acid sequence of the immunising peptide: MLKQSNERRWSLSYKPWSTPETEDVPNTGSNQHRRSICSLGARTGSQASIAPQWTEGNYNYYIEEDEDCGEEGEWKDDLAEENQKAECLTSLLDGHNDTPAQMSTLKVNVGGHSYLLECCELANYPKTRLGRLATSTTRRGQLGLCDDYEAQTDEYFFDRDP.

For retina whole mounts, the preparation and immunostaining of retinas from Kcnv2^E151X/E151X^ (*n* = 3) and Kcnv2^WT/E151X^ (*n* = 3) mice were performed as described in [21].

### 2.5 Imaging and Data Analysis

Stained sections were imaged using a Zeiss LSM 710 confocal microscope (Zeiss, Oberkochen, Germany). Laser power, photomultiplier and digital gain, and pinhole size were kept constant for all samples belonging to the same experimental group to ensure uniformity and comparability of acquired images. The obtained images were further processed and analysed using ImageJ software (National Institute of Health, Bethesda, MD) [22]. This included global adjustment of brightness and contrast to optimise visualisation. GFAP‐positive fibrils were measured in ImageJ with the Segment tool, drawing along the line of each continuous GFAP‐labelled segment from its visible origin at the ganglion cells level to its terminal tip. For outer nuclear layer thickness, we manually counted the number of contiguous rows of photoreceptor nuclei per field of view.

### 2.6 Protein Biochemistry and Western Blots

For protein biochemistry experiments, mice were sacrificed as described above. The retinas of Kcnv2 WT (*n* = 3), Kcnv2^WT/E151X^ (*n* = 3) and Kcnv2^E151X/E151X^ (*n* = 3) mice were dissected in ice‐cold PBS and transferred into Precellys lysing tubes (Bertin Technologies, Montigny‐le‐Bretonneux, France) containing 50 μL lysis buffer (1% triton X‐100, 300 mM NaCl, 0.4% sodium deoxycholate) and 0.5 μL protease inhibitor cocktail (Roche, Basel, Switzerland). The tissue was homogenised using the Minilys system and incubated with constant agitation for 30 min at 4°C and then centrifuged. The supernatants were transferred to fresh tubes and the total protein concentrations were determined using the BCA method (Pierce BCA Protein Assay Kit, Thermo Fisher Scientific). Absorbance was measured at 562 nm using a NanoDrop OneC spectrophotometer. Each measurement was performed in duplicate. Protein concentrations were calculated based on standard curves. Equal amounts of protein (25 μg per sample) were mixed with sample loading buffer and incubated at room temperature for 30–60 min prior to electrophoresis. Samples, along with a molecular weight marker (Colour Prestained Protein Standard, Broad Range, 10–250 kDa; New England Biolabs, Ipswich, MA, USA), were separated by sodium dodecyl sulphate–polyacrylamide gel electrophoresis (SDS‐PAGE) on 10% gels. Proteins were transferred to nitrocellulose membranes using a wet transfer system. Membranes were blocked in 5% blotting‐grade milk prepared in PBS containing 0.1% Tween‐20 (PBST). Primary antibody incubation was performed overnight at 4°C with rabbit anti‐KCNV2 (1:1000) and mouse anti‐GAPDH (1:1000) diluted in blocking buffer. After washing, membranes were incubated for 1 h at room temperature with secondary antibodies: IRDye 800CW goat anti‐rabbit (1:6000, LI‐COR) and IRDye 680RD goat anti‐mouse (1:10000, LI‐COR) and imaged using the ChemiDoc MP Imaging System (Bio‐Rad Laboratories, Hercules, CA, USA). Band intensities were quantified using ImageJ software. KCNV2 signals were lane‐wise normalised to GAPDH to account for potential variation in protein loading.

### 2.7 Statistics

Statistical analysis and data visualisation were performed using ggplot2 [23], dplyr, readxl emmeans, tidyr, cowplot, ggpubr packages in R [24] (version 4.3.2). For each stimulus intensity, differences in amplitudes and or times among the three genotype groups were assessed using a one‐way analysis of variance (ANOVA) followed by pairwise comparisons between groups using Tukey's post hoc test. Adjusted *p* values were used to determine statistical significance, indicated as follows: *p < 0.05, **p < 0.01, ***p < 0.001.

### 2.8 Electroretinogram Recordings

Full‐field electroretinogram recordings were performed following in‐house standards [25]. Specifically, scotopic and photopic ERG recordings were performed in KCNV2^E151X/E151X^ and KCNV2^WT/E151X^ littermates and C57BL/6J WT mice of both sexes at the age of 9–12 weeks, in accordance with the ISCEV standards [20]. The mice were dark‐adapted for at least 20 min before the experiment. The recordings were performed under shallow general anaesthesia, which was induced using 5% isoflurane in 2 L/min O_2_ and maintained using 1.6%–1.8% of isoflurane in 0.8 L/min O_2_, respectively. Pupils were dilated by local application of a drop of Tropicamide 0.5% (Mydriaticum Stulln, manufacturer) and Phenylephrine 5% (Neosynephrin‐POS 5%, manufacturer). Oxybuprocaine hydrochloride (4 mg/mL) was installed to the eye before placing of the electrodes. ERG recordings were performed using CELERIS ERG system (Diagnosys LLC, Lowell, MA, USA), which employs an integrated light guide stimulator and electrode placed on each eye and a ground electrode attached to the tail. For rod response measurement, light stimuli of flash energies ranging from 0.001 to 1 cd × s/m^2^ were presented to the dark‐adapted mice, whereas for cone response isolation, light flashes of intensities ranging from 1 to 10 cd × s/m^2^ and frequencies of 10 and 25 Hz were presented on a constant 30 cd/m^2^ background after > 8 min of adaptation. Data acquisition was performed using the Espion software (Diagnosys) and digitised at 2 kHz. ERG waveforms were analysed to measure various parameters, such as the amplitude and latency of the a‐ and b‐waves.

## 3 Results

### 3.1 Generation of a Novel Mouse line Harbouring the E151X Mutation in the Kcnv2 Gene

In order to develop a mouse model that would genetically more closely resemble the majority of cases of KCNV2‐related retinopathy, we generated a mouse line carrying the early truncating E151X mutation (Figure 1A, c.583G>T), orthologous to the human E143X, in the Kcnv2 gene using CRISPR/Cas9 genome editing. The introduced mutation created a novel *BfaI* restriction site, enabling efficient genotyping of this mouse line by PCR amplification followed by *BfaI* digestion (Figure 1B–D) Two founder animals were identified and back‐crossed to C57Bl/6J mice for at least four generations before the line was used in experiments. Offsprings from these matings were fertile and viable regardless of genotype or sex.

**FIGURE 1:**
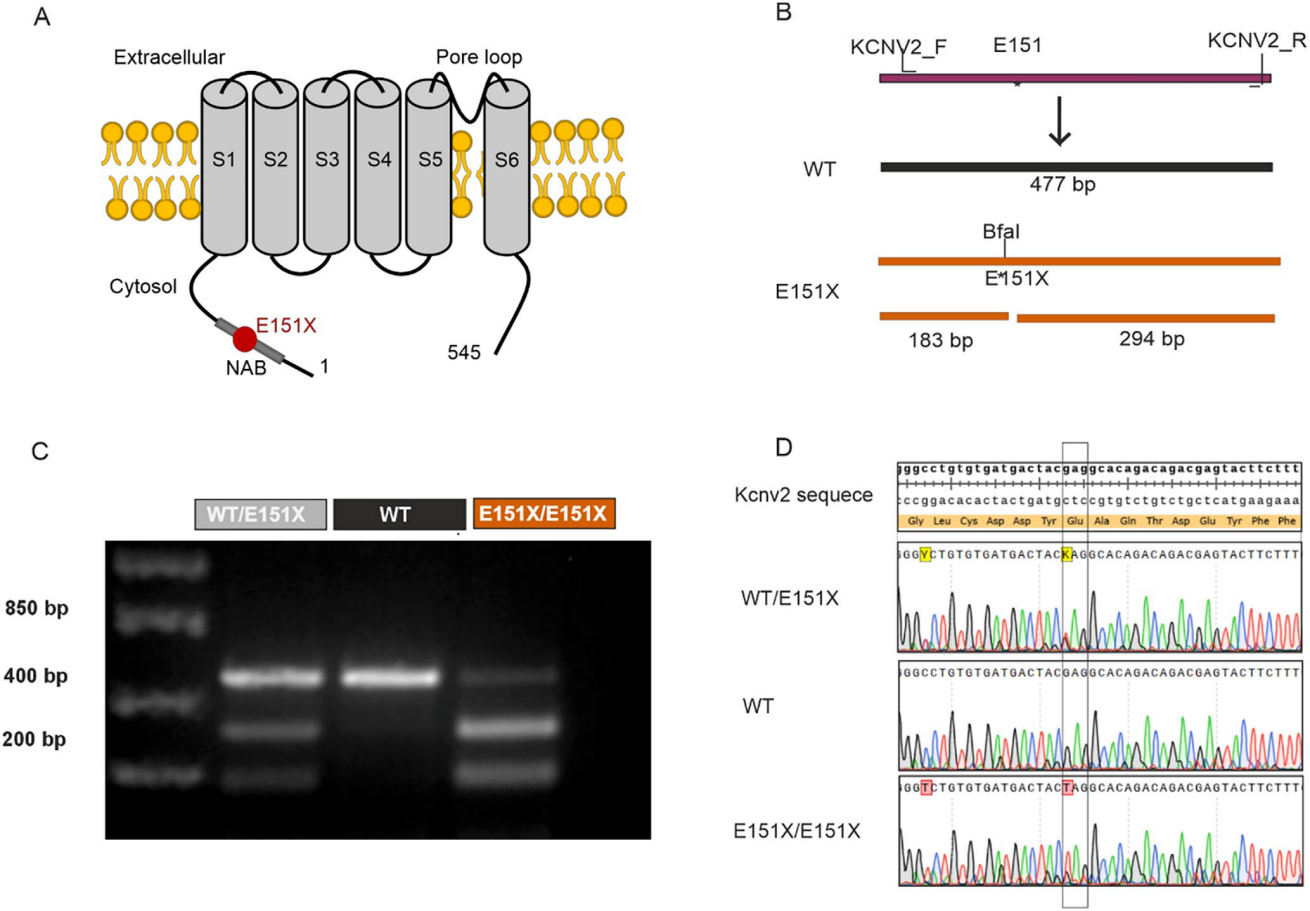
Generation and validation of the mutant mouse line using CRISPR/Cas9 and Sanger sequencing. (A) Schematic representation of the mutation's localisation in the Kcnv2 tetramerisation domain (NAB). (B) Genotyping strategy using PCR amplification followed by BfaI digestion. The restriction site is introduced only in the mutant allele, allowing selective cleavage. (C) Representative genotyping gel showing WT, heterozygous (Kcnv2^WT/E151X^) and homozygous mutant (Kcnv2^E151X/E151X^) animals and their corresponding band patterns. (D) Exemplary Sanger sequencing chromatograms of WT, Kcnv2^WT/E151X^ and Kcnv2^E151X/E151X^ mice.

### 3.2 K_v_8.2 Is Absent in the Kcnv2E151X Mouse

Employing immunohistochemistry on retinal cryosections and Western blot, we confirmed that the full‐length protein was absent in these mice. Immunohistochemical staining of retinal cryosections resulted in distinct staining patterns in the Kcnv2^WT/WT^ tissues compared to the Kcnv2^E151X/E151X^ ones. In the WT samples, K_v_8.2 immunoreactivity was specifically localised to the photoreceptor inner segments, in full agreement with previous reports. In the Kcnv2^E151X/E151X^ retinal sections, we detected a complete absence of staining for K_v_8.2 in two mice and a weak residual K_v_8.2 signal in two other Kcnv2^E151X/E151X^ mice (Figures 2A and S1). The residual signal can be possibly explained by the antibody recognising the truncated K_v_8.2 protein, as the immunising peptide used for antibody generation encompassed AA 1–163 of the K_v_8.2's N‐terminal cytoplasmic region, which is still present in the truncated protein. This faint signal was observed at the very tip of the inner segment of photoreceptors, but was markedly less intense compared to the WT tissues. To further evidence the absence of full‐length K_v_8.2 in the Kcnv2E151X mouse model, we performed Western blot analysis on retinal lysate samples from Kcnv2^E151X/E151X^ and WT littermates (*n* = 3 each). Western blot analysis revealed the presence of full‐length K_v_8.2 (65 kDa) in retinas from the WT mice. In contrast, no corresponding band was detected in the Kcnv2^E151X/E151X^ retinal lysates (Figure 2B). Taken together, these results confirm the effectiveness of the knockout strategy and validate the loss of full‐length K_v_8.2 in our mouse line.

**FIGURE 2:**
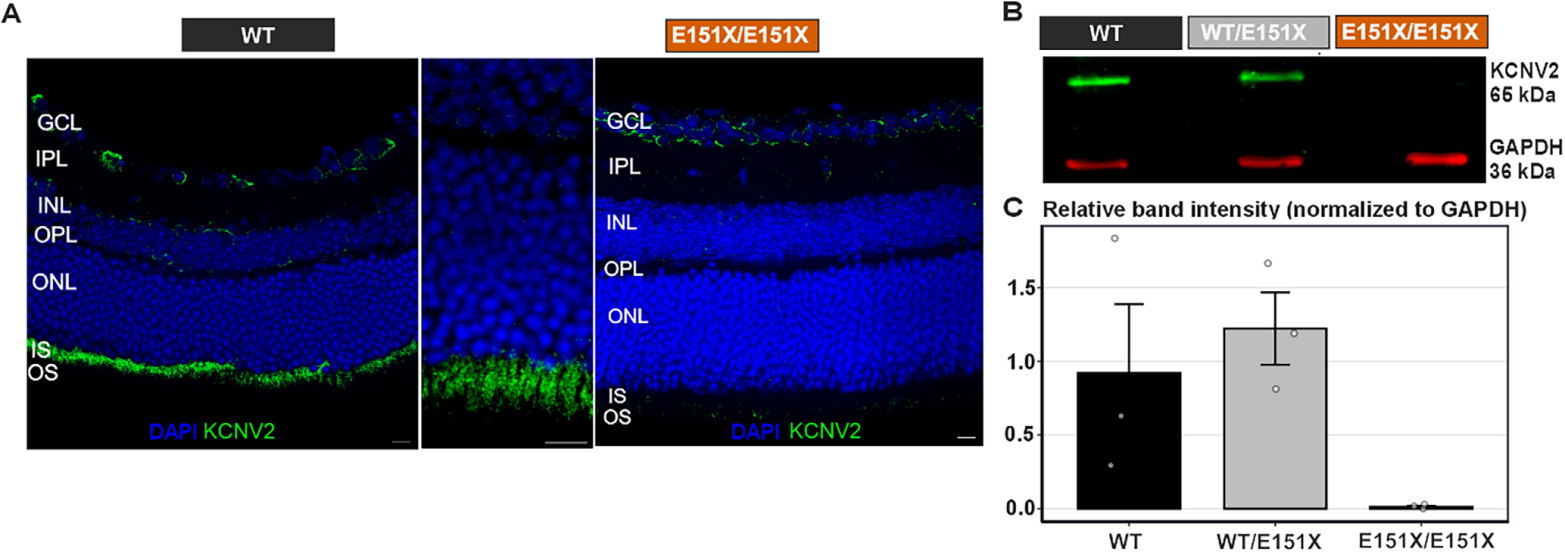
Expression of K_v_8.2 in WT, Kcnv2^WT/E151X^ and Kcnv2^E151X/E151X^ retinas. (A) Immunohistochemistry showing K_v_8.2 localisation in retinal sections from WT (left panel), WT‐zoomed (middle panel), and Kcnv2^E151X/E151X^ mice (right panel). K_v_8.2 is observed in the IS of the WT retina, while signal is absent in Kcnv2^E151X/E151X^ samples, confirming the loss of expression. (B) Representative Western blot of K_v_8.2 in retinal lysates from WT, Kcnv2^WT/E151X^ and Kcnv2^E151X/E151X^ littermates. GAPDH was used as a loading control. A 65 kDa band corresponding to K_v_8.2 was only detected in WT and heterozygous samples. (C) Quantification of K_v_8.2 band intensity normalised to GAPDH, showing comparable levels of protein expression in WT and Kcnv2^WT/E151X^ mice and complete absence of the protein in homozygous samples. Data are presented as mean ± SEM. *n* = 3 mice for each genotype. The full, uncropped blot image is given in Figure S2.

### 3.3 Kcnv2E151X Mice Show a Loss of Cones and Reduction in Photoreceptors Layers

To explore to what extent our new mouse line resembled the human disease phenotype, we first quantified cone loss at different ages by the means of quantifying cone arrestin (CArr) immunopositive somata in the outer nuclear layer. Kcnv2^E151X/E151X^ mice exhibited a continuous decline in CArr positive soma with age (slope = −0.18 CArr^+^ somata/week/field of view [FoV], *p* = 0.013), while no such decline could be observed in WT littermates (slope = 0.00 CArr^+^ somata/week/FoV, *p* = 0.6, Figure 3). In addition to cone loss, a loss of photoreceptor cells in general in homozygous mutant mice was also observed, as evidenced by a decrease in rows of (DAPI^+^) nuclei in the ONL. Kcnv2^E151X/E151X^ mice lost −0.11 rows/week/FoV (*p* = 0.056) while the loss in WT littermates was substantially less pronounced (−0.06 rows/week/FoV, *p* = 0.185, Figure 3). We were not able to assess rod photoreceptor counts separately. It appears therefore unlikely that the marked decrease in rows of ONL nuclei would be due to cone loss alone and is likely reflecting additional rod loss in our Kcnv2^E151X^ mouse model.

**FIGURE 3:**
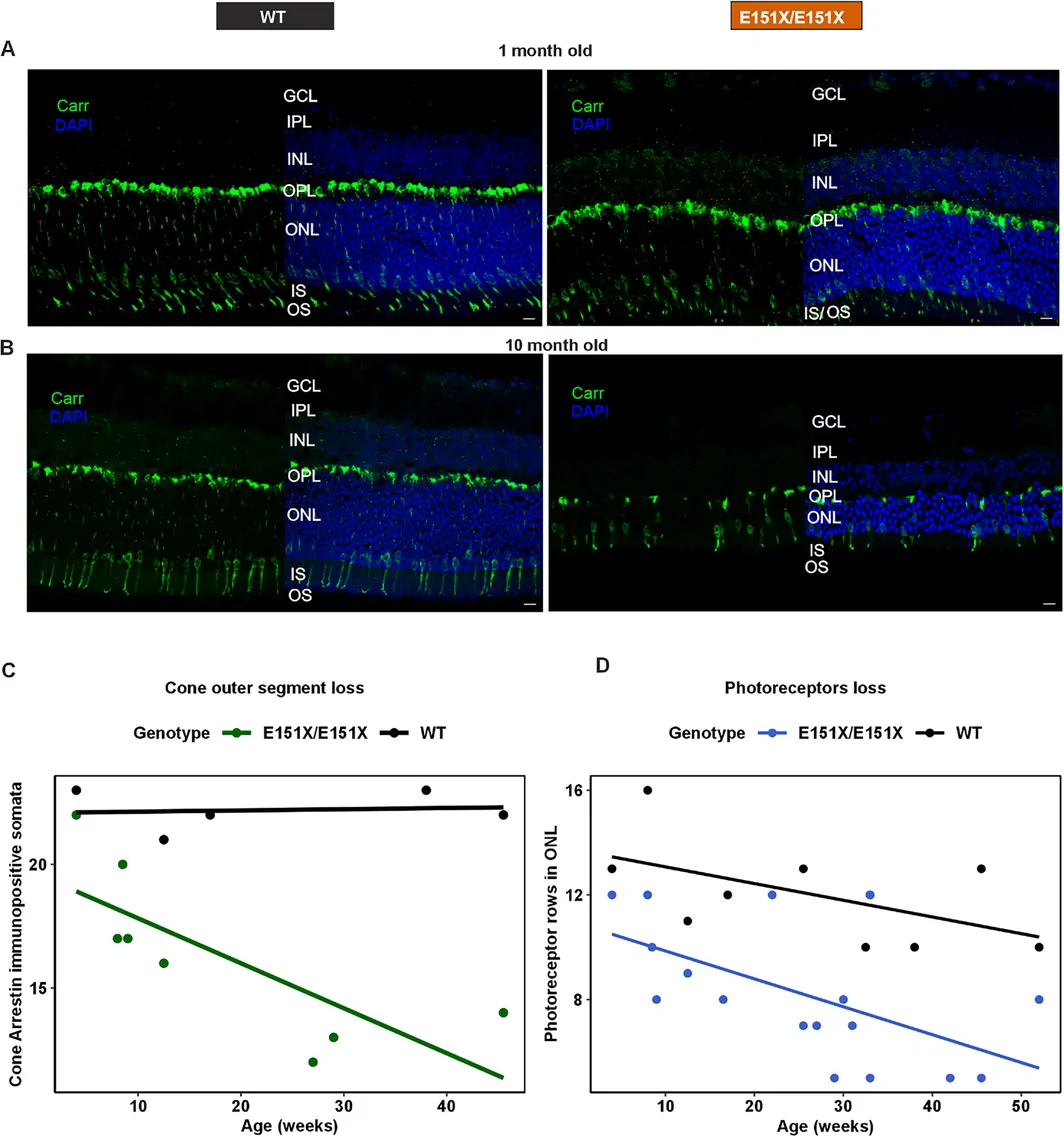
Progressive photoreceptor loss in Kcnv2^E151X/E151X^ mice. (A) Immunohistochemistry for cone arrestin in 1‐month‐old WT (left) and Kcnv2^E151X/E151X^ (right) retinas. Cone arrestin‐positive cells are readily observed in both genotypes, with no marked deficit in the K_v_8‐mutant retinas. (B) Cone arrestin staining in 10‐month‐old WT and Kcnv2^E151X/E151X^ retinas. Kcnv2^E151X/E151X^ mice show a clear reduction in cone arrestin‐positive cells compared to age‐matched WT controls, indicating cone loss over time. (C) Quantification of cone arrestin‐positive somata in the outer retinal layer across age. WT mice maintain stable cone arrestin expression, whereas Kcnv2^E151X/E151X^ mice exhibit a progressive decline, consistent with age‐dependent cone loss. Each dot in the graph represents one mouse. Matching results could be obtained when quantifying cone arrestin‐positive somata on retinal flat mounts in 36‐weeks‐old mice (Figure S3). (D) Outer nuclear layer (ONL) thickness measured across age shows progressive ONL thinning in Kcnv2^E151X/E151X^ mice, reflecting overall photoreceptor degeneration. WT mice show minimal ONL thinning over the same period.

### 3.4 GFAP Is Highly Expressed in Kcnv2E151X Mice

Reactive gliosis is commonly observed in retinal degenerations [26] and goes along with remodelling of the internal limiting membrane [27, 28] that alters the ability of certain adeno‐associated viruses (AAV) to penetrate the retina. We therefore used glial fibrillary acidic protein (GFAP) immunoreactivity as a marker for Müller glial activation. In Kcnv2^E151X/E151X^ mice, Müller glial cell activation was assessed by measuring the ratio of GFAP fibril length to retina thickness (Figure 4). The GFAP fibril length to retina thickness ratio was consistently higher in the mutant mice compared to the WT ones across various ages, with a noticeable difference starting from as early as 4 weeks old. Fibril length was mostly stable over ages and showed a minimal downward trend in Kcnv2^E151X/E151X^ mice (slope = 0.003, *p* < 0.001). In contrast, no relevant activation of Müller glial cells could be observed in WT mice.

**FIGURE 4:**
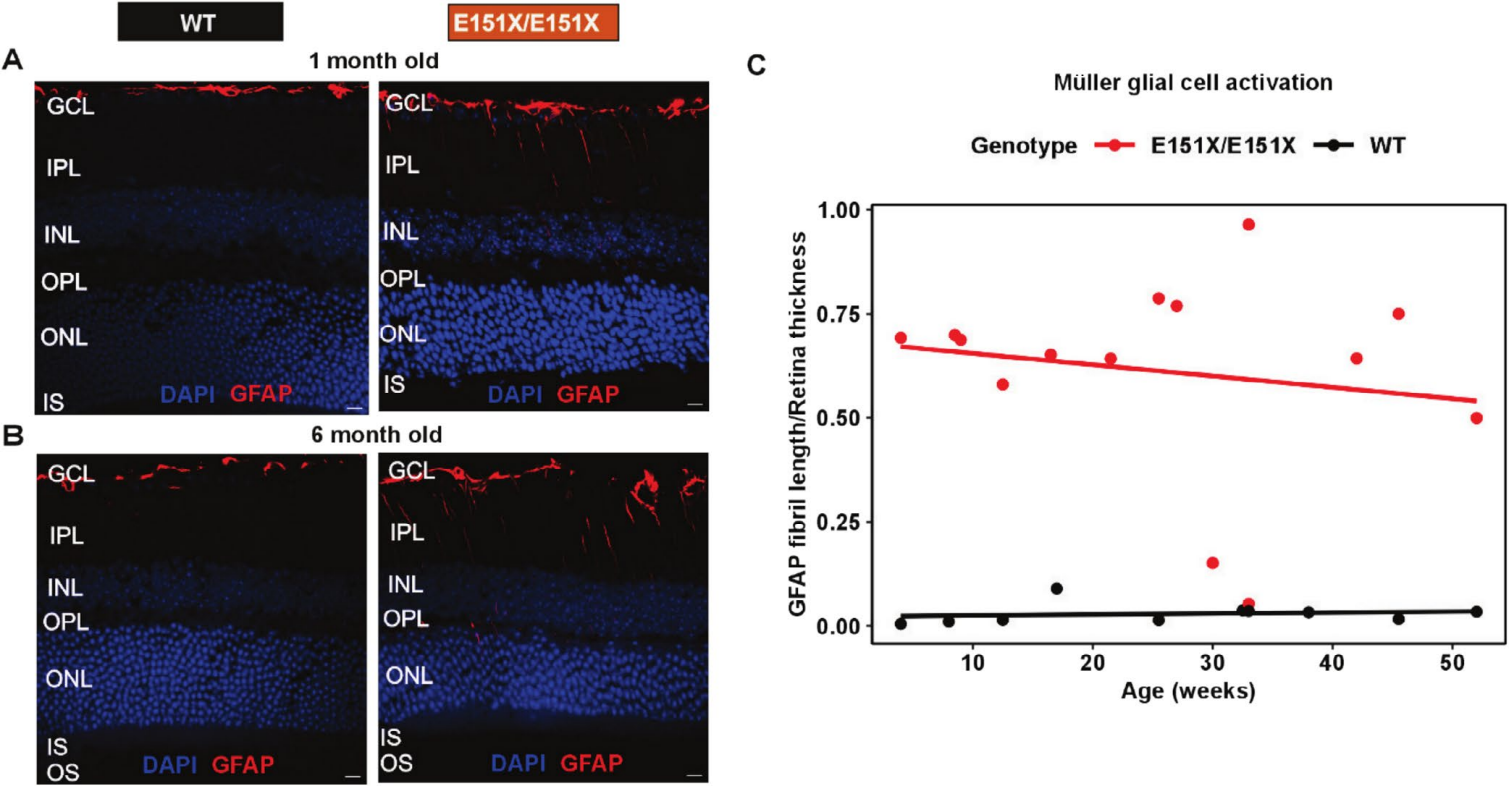
Müller glial activation in the Kcnv2E151X mouse model. (A) Representative retinal sections from 1‐month‐old WT and Kcnv2^E151X/E151X^ mice stained for GFAP. At 1‐month, in mutant mice GFAP immunoreactivity is already largely present as fibrils extending towards the outer retina, whereas stainings in age‐matched WT mice no detectable glial activation. (B) GFAP staining in 6‐month‐old WT and Kcnv2^E151X/E151X^ retinas. Kcnv2^E151X/E151X^ mice display robust GFAP‐positive radial fibrils extending towards the ONL, whereas in WT retinas GFAP localisation remains at the GCL. (C) Quantification of GFAP fibril length normalised to retinal thickness as a function of age (weeks) in WT (black dots) and Kcnv2^E151X/E151X^ (red dots) mice. Kcnv2^E151X/E151X^ mutants show a significant upregulation in GFAP beginning at 4 weeks and persisting through more than 50 weeks of age, indicative of an early and sustained Müller glial activation, whereas WT values remain at baseline values. Each point represents one retina.

### 3.5 Visual Function Largely Resembles Kcnv2‐Associated Retinopathy Patients' Responses

To evaluate the electrophysiological characteristics of our mouse line, we performed full‐field electroretinogram recordings in six Kcnv2^E151X/E151X^, seven WT and six Kcnv2^WT/E151X^ aged between 9 and 12 weeks. The rod response was assessed by measuring the amplitudes of a‐wave and b‐wave components under dark‐adapted conditions at 0.001, 0.01, 0.1 and 1 cd × s/m^2^ stimuli intensities. The a‐wave amplitudes were significantly smaller in Kcnv2^E151X/E151X^ mice compared to WT, when stimulated by 0.1 and 1 cd × s/m^2^ flashes. The heterozygous mice showed a phenotype that mostly resembled that of the WT mice, though, there was a slight, non‐significant, reduction in amplitudes (Figure 5B). When contemplating the b‐waves, the amplitudes in mutant mice were substantially and significantly lower than for WT mice upon dim flashes, while from 0.01 cd × s/m^2^ on, these increased to levels indifferent and sometimes exceeding those observed in WT mice (Figure 5C). We further analysed the timing of the rod‐pathway responses by examining the time to peak (implicit time) of the a‐ and positive b‐wave under scotopic conditions. The Kcnv2^E151X/E151X^ mice displayed a delayed a‐wave implicit time compared to WT mice, indicating a slower initial response of the rod photoreceptors to the light flashes (Figure 5D). Moreover, the a‐wave troughs were broader in K_v_8.2^E151X/E151X^ mice, as opposed to the WT and heterozygous ones (Figure 5A) Additionally, the b‐wave implicit time was significantly prolonged in Kcnv2^E151X/E151X^ mice relative to WT mice, reflecting delayed post‐phototransduction signalling in the rod pathway across an increasing light stimulus energy (Figure 5E). To evaluate cone function, we measured the photopic ERG responses under light‐adapted conditions (Figure 6). The cone‐mediated a‐wave amplitudes were reduced in Kcnv2^E151X/E151X^ mice compared to WT mice. The responses were significantly smaller at 3 and 10 cd × s/m^2^ when compared to the WT group and only at 3 cd × s/m^2^ when compared to the heterozygous group (Figure 6A,B). The decrease in amplitudes was also observed for the b‐waves, which were markedly lower in the Kcnv2^E151X/E151X^ group from 3 cd × s/m^2^ onwards, compared to both—WT and heterozygous littermates. Analysis of oscillatory potentials revealed significant differences in OP amplitudes of Kcnv2^E151X/E151X^ mice (Figure 7). Quantification of OP1, OP2 and OP3 at 0.1 cd × s/m^2^ and analysis by multivariate linear regression a large reduction in OP amplitude in homozygous Kcnv2^E151X/E151X^, compared to both WT and heterozygous mice (Figure 7B).

**FIGURE 5:**
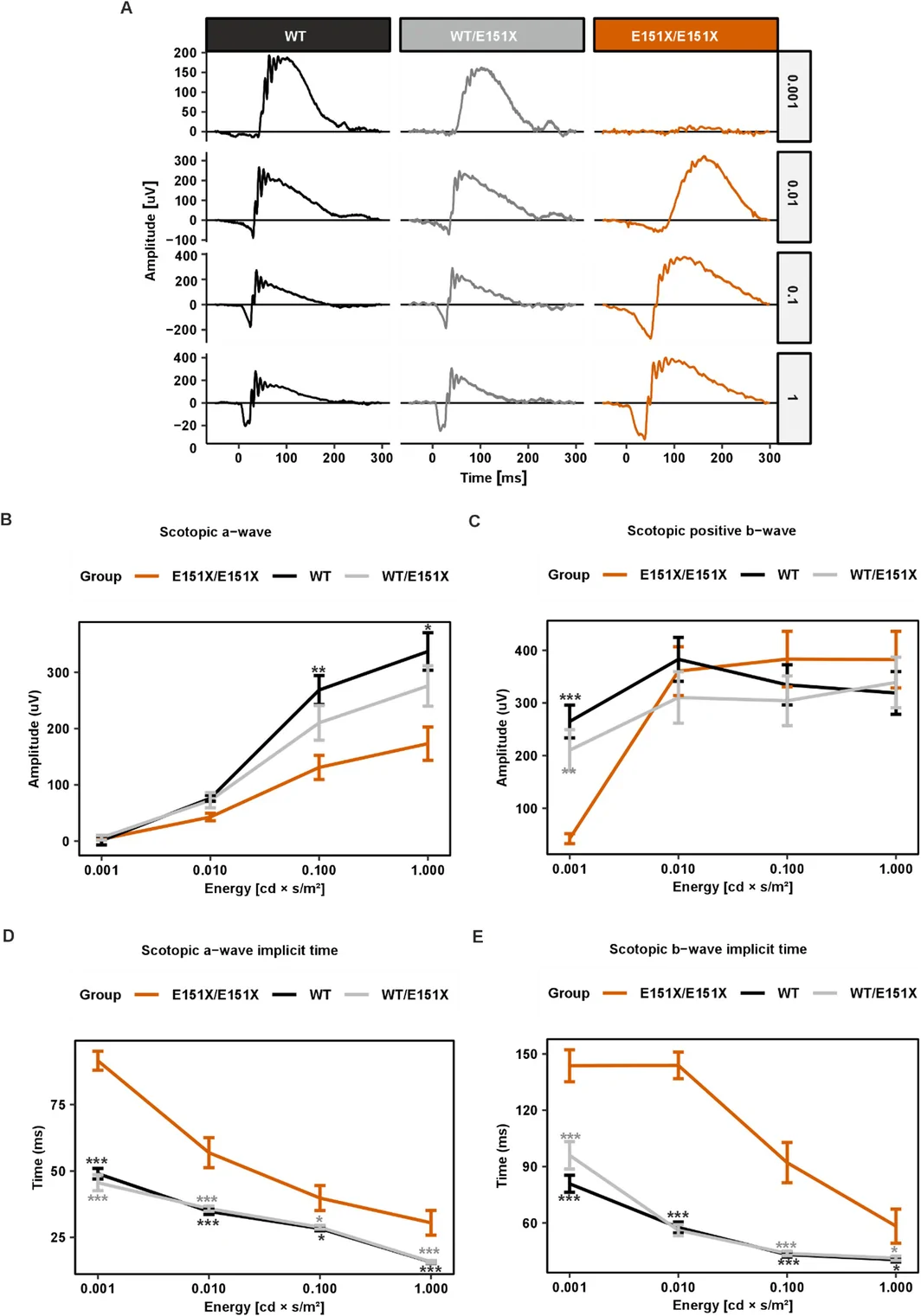
Scotopic ERG responses in WT, Kcnv2^WT/E151X^ and Kcnv2^E151X/E151X^ mice. (A) Representative ERG traces for each genotype in response to increasing flash energies (0.001, 0.01, 0.1 and 1 cd × s/m^2^), illustrating stimulus‐dependent response characteristics. (B) Amplitude of the scotopic a‐wave recorded from wild‐type (WT), heterozygous (Kcnv2^WT/E151X^) and homozygous (Kcnv2^E151X/E151X^) mice. (C) Amplitude of the scotopic b‐wave. (D and E) Implicit time of the scotopic b‐wave and a‐wave, showing differences in response kinetics across genotypes. Data for (A)–(D) are presented as mean ± SEM. Statistical significance was assessed using one‐way ANOVA with post hoc Tukey test. *n* = 6–7 eyes per genotype. *p < 0.05, **p < 0.01, ***p < 0.001.

**FIGURE 6:**
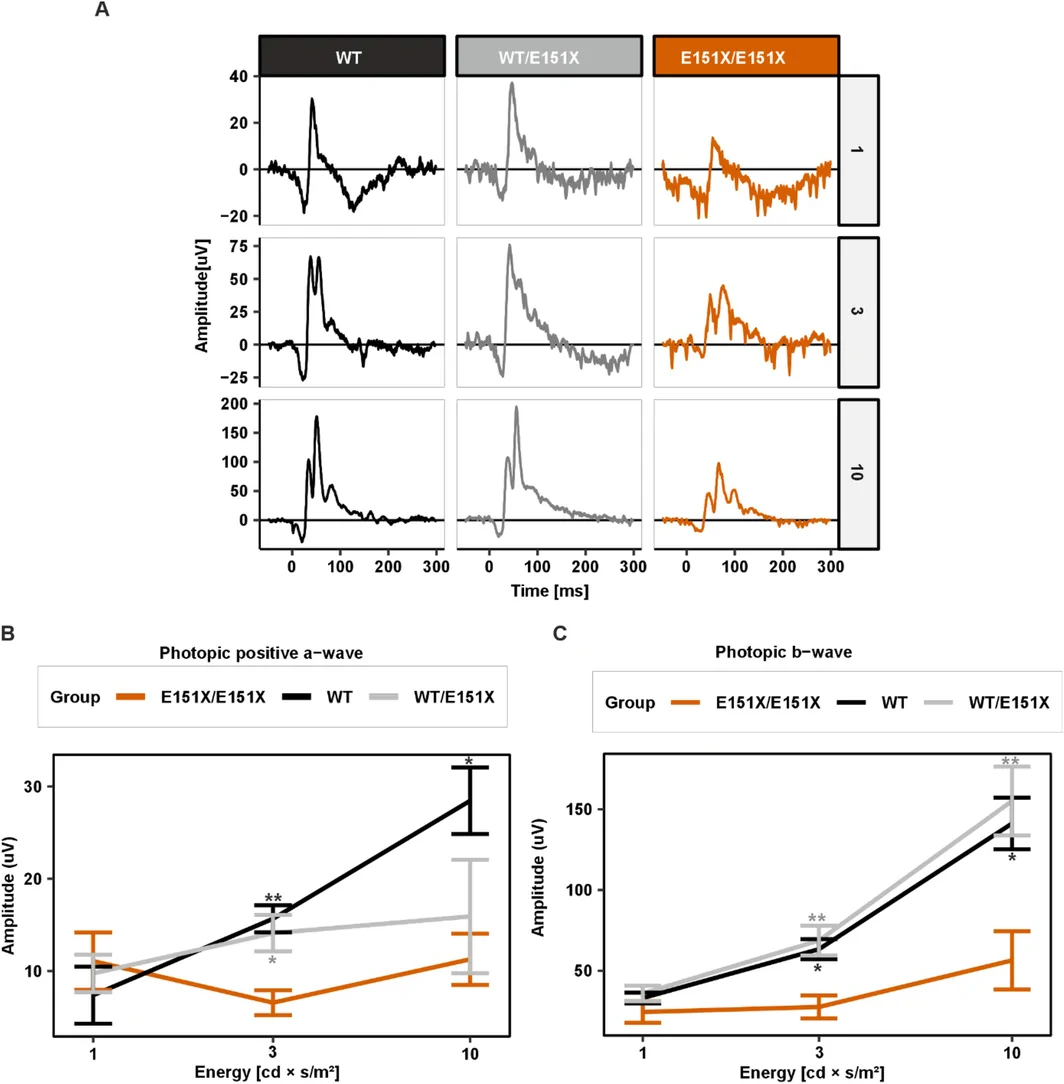
Photopic ERG responses in WT, Kcnv2^WT/E151X^ and Kcnv2^E151X/E151X^ mice. (A) Representative photopic ERG traces for each genotype in response to increasing flash energies (1, 3 and 10 cd × s/m^2^). (B) Amplitude of the photopic a‐wave recorded from WT, Kcnv2^WT/E151X^ and Kcnv2^E151X/E151X^ mice under light‐adapted conditions, reflecting cone photoreceptor activity. (C) Amplitude of the photopic b‐wave for the same genotypes, indicating bipolar cell function integrity in the cone pathway. Data for (B) and (C) are shown as mean ± SEM. Statistical comparisons were performed using one‐way ANOVA with Tukey's post hoc. *n* = 5–7 eyes per group; *p < 0.05, **p < 0.01, ***p < 0.001.

**FIGURE 7:**
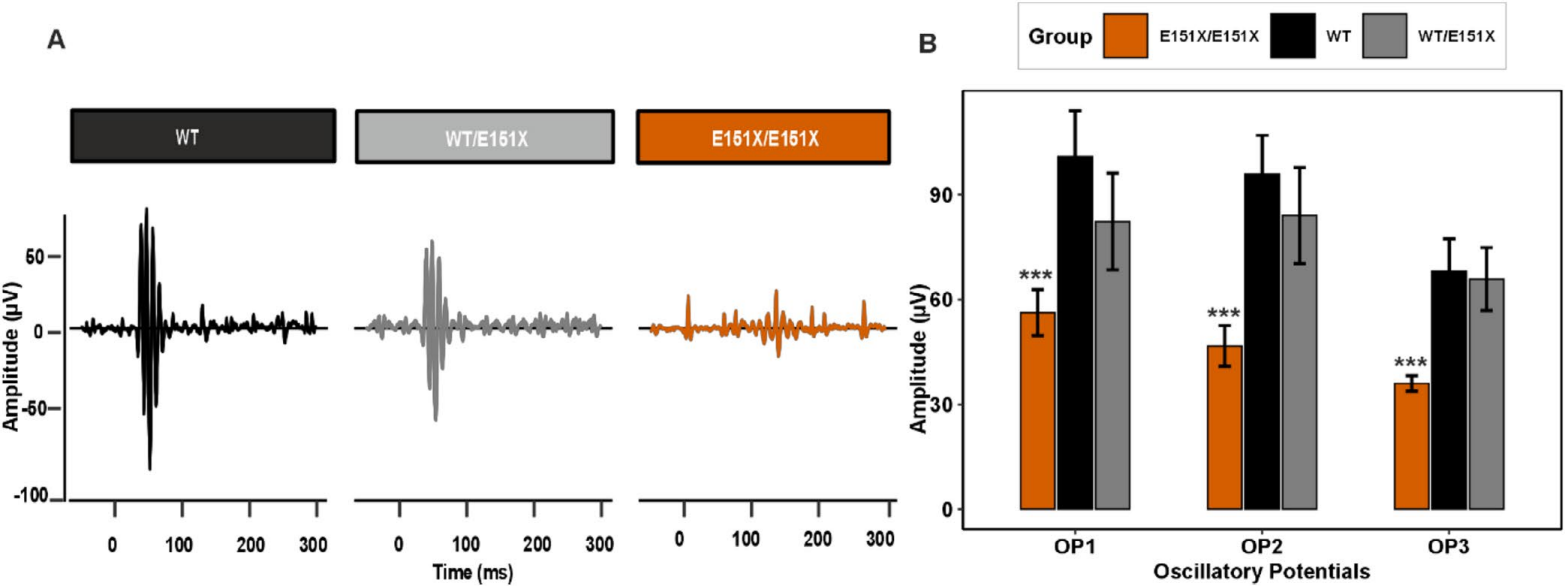
Representative oscillatory potential (OP) traces and quantification of OP amplitudes in WT, Kcnv2^WT/E151X^ and Kcnv2^E151X/E151X^ mice. (A) Representative oscillatory waveforms recorded from WT, Kcnv2^WT/E151X^ and Kcnv2^E151X/E151X^ mice, illustrating the characteristic reduction in OP amplitude in the Kcnv2^E151X/E151X^ group. (B) Quantitative comparison of OP1, OP2 and OP3 amplitudes across the three genotypes at 0.1 cd × s/m^2^. Data are presented as mean ± SEM. Statistical comparisons were performed with two‐way ANOVA following multivariate regression with WT and OP1 as reference levels. Reduction of OP amplitudes was significant in homozygous mice compared to both wild‐type and heterozygous mice across all three OPs assessed. *n* = 5–7 eyes per group.

### 3.6 Interventional Trials Are Feasible Using Similar Functional Outcome Measure in Mice and Man

Having observed profound similarities between the human disease and the phenotype of the Kcnv2^E151X^ mouse, we reasoned that this mouse model might well be suited as a starting point for assessing efficacy of a hypothetical new treatment approach. We reasoned that given the structural stability of the rod system in particular and the characteristic electrophysiological changes, dark‐adapted ERG metrics would be best suited for proof of concept. As nyctalopia is a common symptom in KCNV2‐associated retinal dystrophy patients [15], it is moreover comparably straightforward to argue the clinical relevance of an improvement in dark‐adapted ERG metrics towards a regulator. In this regard, having a single (or analog) metric that could be used in mouse and man might additionally facilitate translation.

## 4 Discussion

In the present work we report on the generation and characterisation of the Kcnv2^E151X^ mouse, carrying the orthologue of the common human E143X mutation. We found that the retinal phenotype of Kcnv2^E151X^ mirrors key characteristics of KCNV2‐related retinopathy. Specifically, the mice lack expression of full‐length K_V_8.2, undergo progressive cone loss and display the characteristic ERG pattern with delayed scotopic responses, disproportionate b‐wave growth, and severely reduced photopic signals. These concordances indicate that the model accurately reflects human disease biology.

Two other mouse models for KCNV2‐related retinopathy have been previously reported [29, 30]. As compared to those models, the mutation induced in the model described herein induces an early truncation in the middle of the NAB domain as opposed to the (virtually) complete knock‐out obtained in the previous models [29, 30]. E143X is the most common single variant associated with KCNV2‐related retinopathy, accounting for 12.6% of all mutations linked to the disease [15]. Thus, on a genetic level, our model closely resembles this common disease‐causing variant. Mutations within the NAB, which is relevant for efficient heteromerisation of compatible K_V_ subunits [29] are overall common including several early truncation variants and 35.7% of all missense variants associated with the disease [15]. Under the general assumption that an early stop on gene level would result in an early truncation protein in mice as in man, we expect that a shortened protein is produced lacking most functional domains including half of the NAB domain and all transmembrane domains. We can therefore assume that the model presented herein may be closely representative for a large proportion of affected individuals. Yet, it needs to be noted that we were not able to evidence this shortened protein, e.g., by Western blot analysis.

Smith et al. had previously shown in cell line experiments that mechanistically distinct types of mutations may occur. Mutations in proximity to the NAB domain lead to production of homomeric K_V_2 channels that show currents that are larger and biophysically distinct when compared to the physiological K_V_8.2–K_V_2 heteromers (specifically: left‐shifted voltage activation, faster inactivation). Pore mutations lead to the production of non‐functional K_V_8.2–K_V_2 heteromers and a small portion of K_V_2 homomers, resulting in, most notably, a profound reduction in current amplitudes [16]. Although that study had only explored two mutations *neighbouring* the NAB, their data suggest that early mutations, like E151X (equalling the human E143X) might be susceptible to gene replacement therapy approaches or pharmacological strategies that primarily reduce current K_V_2‐mediated current amplitudes. Notably, experiments on the functional consequences of the E151X mutation have yet not been published. It is also unclear what the consequence of the expression of aberrant Kcnv2–E151X protein is. Some Kcnv2–E151X mRNA might undergo nonsense‐mediated decay [30]. Yet, at least to some extent, Kcnv2–E151X mRNA would also be translated into truncated protein. This truncated protein would then contain a partial NAB domain, possibly compatible towards K_V_2 and thereby potentially resulting in intracellular accumulation or even sequestering K_V_2 partners. The fact that we detected some mislocalised Kcnv2‐immunoreactivity in some Kcnv2^E151X/E151X^ mice when using antibodies directed against the N‐terminus is suggestive for a low degree of Kcnv2–E143X protein production—at least in some mice. Yet, as a note of caution, we did not detect the expected a ~13 kDa band in our western blots (using a different antibody and having usually weaker signal‐to‐noise ratios as compared to IHC). If it could be confirmed that relevant amounts of truncated protein reside in the cell, how this contributes to the disease process could be studied in the mouse line presented herein, possibly by structural comparison to the available full knock‐out lines.

To date, there is robust evidence that K_V_8.2–K_V_2 heterotetramers substantially contribute to I_K,X_, including by electrophysiology and co‐immunoprecipitation from native tissue [6]. Due to its similarity to the neuronal m‐current the original hypothesis however had been that K_V_7 channels might contribute to I_K,X_ [8]. Generally consistent with this observation another study found an approximately 30% reduction in I_K,X_ following Kv7 blocking; Yet, because, this reduction did not reach statistical significance, the authors concluded that there was no relevant contribution of K_V_7–I_K,X_ [6]. Nevertheless, As K_V_7 channels are a proven pharmacological target, e.g., in the context of cardiovascular disease [31], their possible contribution to I_K,X_ still deserves further evaluation and any possible contribution to I_K,X_ could be tested comparing the effects of K_V_7 modulators in the Kcnv2^E151X^ vs. wild‐type mice.

The Kcnv2^E151X^ mouse phenocopies key characteristics of the human disease [15]. Specifically including dark‐adapted b‐waves that start subnormal at low and show accelerated progression towards higher stimulus energies. Also, the broadened a‐wave trough and delayed b‐wave peaks are present in the Kcnv2^E151X^ mouse. The supernormal b‐wave at high stimulus intensities that has initially been namesake for the disease can only be observed occasionally in the Kcnv2^E151X^ mouse and on average remains on the upper margin of normality—just as in man [2, 15]. Notably, in this mouse model we observed a pronounced reduction in photoreceptor layer thickness over time, which cannot be explained by a loss of cones alone, as these account only for 2.8% of the total number of photoreceptors in the mouse retina [32] Instead, we assume this represents concomitant rod loss. This is at least in part in contrast to human disease, where the rod‐dominated peripheral retina remains widely intact even in advanced disease stages [15, 33]. Our observation that rods degenerate over the disease course in mice is consistent with the observations made in the other two available mouse models [30, 34], suggesting that this is truly a difference between the human and the murine disease phenotype (as opposed to a mouse model artefact). Inamdar et al. highlight that in human the cone‐exclusive fovea is surrounded by a ring of high rod density and hence structural macular affection—the hyperautofluorescent ring or even atrophy—as sometimes seen in KCNV2‐related retinopathy patients would necessarily involve rod‐rich areas [30]. Yet, it needs to be taken into consideration that the highest rod densities are found clearly more eccentric than the structural alteration seen in KCNV2‐related retinopathy [15, 35, 36]. We rather see parallels between the cone‐to‐Muller cell ratio and the location of the hyperautofluorescent ring [37]. In any case, rod loss in the mouse model exceeds that observed in humans. Potential reasons may include distinct clearance of extracellular potassium by Muller cells, which might be least effective in the human (para‐)fovea [34]. Or a specific susceptibility of cones in humans: Contrary to mice, human cones express a second K_V_2 isoform, K_V_2.2, which is absent in rods. Thus, in humans I_K,x_ has a slightly distinct structural basis in rods and cones and the involvement of K_V_2.2 might lead to more unfavousrable electrophysiological changes in these cells in the absence of KCNV2. An alternative hypothesis to consider is that nocturnal mice utilise their rod system more than man, and this might, by some mechanism, lead to a more severe affection of the rods.

Nevertheless, the course of photoreceptor degeneration in the Kcnv2^E151X^ mouse is slow, with cones remaining immunohistochemically detectable until at least 1 year of age (Figure 4). This course temporally mirrors the clinical course in human patients [15, 38], indicating that there is ample room for therapeutic intervention.

One interesting observation is that the physiological phenotype of the retina of heterozygous mice closely, but not entirely, mirrors that of the WT mice, with small differences in amplitudes that are mostly not significant in a statistical context. This observation matches the clinical conclusions that the KCNV2‐related retinopathy is a recessive disease. Yet, they also leave room for speculation on a minor haploinsufficiency phenotype. In this regard, refined electrophysiological protocols might be required to further characterise any such changes.

A particular strength of our report is that we provide longitudinal data for cone and global photoreceptor counts over the first year of life, a time span which would roughly correlate to the 40 years of human life. This information can prove helpful when aiming to model specific disease stages or planning treatment studies. Over the same timespan we also observe Muller glia activation. We observe that modest glia activation occurs early in the disease, but then remains roughly stable over the entire disease course. The latter indicates that no major retinal remodelling or glial seal formation seems to occur. Additionally, while sharing basic similarities with the other Kcnv2 mouse models [30, 34], the model presented herein exhibits some features which parallel the human disease phenotype somewhat closer than those. Particularly, square‐shaped/broaden a‐waves are constantly observed in human upon bright DA stimuli and these are also present in our mouse line while this is not the case in others [34]. Similarly, cone loss is more pronounced in this model than in the others [30, 34].

In summary, we have successfully created and characterised a novel mouse line for the KCNV2‐related retinopathy. This new model very closely resembles the morphological and electrophysiological changes described in human patients. Even though the mouse lacks a macula, the strongly conserved photoreceptor physiology and the matching diagnostic ERG patterns make this model a fit platform for testing treatment options and gaining deeper insight into the pathophysiology of the disease.

## Funding

This work was supported by Deutsche Forschungsgemeinschaft (LI 2846/5‐1, LI 2846/6‐1).

## Conflicts of Interest

The authors declare no conflicts of interest.

## Supporting information


**Figure S1:** Representative retinal sections immunolabelled for Kv8.2 in WT (A) and Kcnv2^E151X/E151X^ mice (B).
**Figure S2:** Raw Western blot for Kcnv2 in retinal lysates from three genotypes.
**Figure S3:** Cone arrestin immunostaining in retinal wholemounts.

## Data Availability

The data that support the findings of this study are available from the corresponding author upon reasonable request.

## References

[1] P. Gouras , H. M. Eggers , and C. J. MacKay , “Cone Dystrophy, Nyctalopia, and Supernormal Rod Responses. A New Retinal Degeneration,” Archives of Ophthalmology 101, no. 5 (1983): 718–724. 10.1001/archopht.1983.01040010718003 6601944

[2] H. Wu , J. A. Cowing , M. Michaelides , et al., “Mutations in the Gene KCNV2 Encoding a Voltage‐Gated Potassium Channel Subunit Cause ‘Cone Dystrophy With Supernormal Rod Electroretinogram’ in Humans,” American Journal of Human Genetics 79, no. 3 (2006): 574–579. 10.1086/507568 PMC1559534 16909397

[3] B. Wissinger , S. Dangel , H. Jägle , et al., “Cone Dystrophy With Supernormal Rod Response Is Strictly Associated With Mutations in KCNV2,” Investigative Ophthalmology & Visual Science 49, no. 2 (2008): 751–757. 10.1167/iovs.07-0471 18235024

[4] J. S. Gill , M. Georgiou , A. Kalitzeos , A. T. Moore , and M. Michaelides , “Progressive Cone and Cone‐Rod Dystrophies: Clinical Features, Molecular Genetics and Prospects for Therapy,” British Journal of Ophthalmology 103, no. 5 (2019): 711–720. 10.1136/bjophthalmol-2018-313278 PMC6709772 30679166

[5] D. H. Sakti , E. E. Cornish , H. Ali , et al., “Natural History and Biomarkers of KCNV2‐Associated Retinopathy,” Clinical & Experimental Ophthalmology 52, no. 5 (2024): 528–544. 10.1111/ceo.14373 38443311

[6] J. Gayet‐Primo , D. B. Yaeger , R. A. Khanjian , and T. Puthussery , “Heteromeric K(V)2/K(V)8.2 Channels Mediate Delayed Rectifier Potassium Currents in Primate Photoreceptors,” Journal of Neuroscience 38, no. 14 (2018): 3414–3427. 10.1523/JNEUROSCI.2440-17.2018 PMC5895036 29483285

[7] G. Czirjak , Z. E. Toth , and P. Enyedi , “Characterization of the Heteromeric Potassium Channel Formed by kv2.1 and the Retinal Subunit kv8.2 in *Xenopus oocytes* ,” Journal of Neurophysiology 98, no. 3 (2007): 1213–1222. 10.1152/jn.00493.2007 17652418

[8] D. J. Beech and S. Barnes , “Characterization of a Voltage‐Gated K+ Channel That Accelerates the Rod Response to Dim Light,” Neuron 3, no. 5 (1989): 573–581. 10.1016/0896-6273(89)90267-5 PMC3858083 2642011

[9] A. O. Khan , M. Alrashed , and F. S. Alkuraya , “'Cone Dystrophy With Supranormal Rod Response' in Children,” British Journal of Ophthalmology 96, no. 3 (2012): 422–426. 10.1136/bjophthalmol-2011-300271 21900228

[10] A. Vincent , T. Wright , Y. Garcia‐Sanchez , et al., “Phenotypic Characteristics Including In Vivo Cone Photoreceptor Mosaic in KCNV2‐Related ‘Cone Dystrophy With Supernormal Rod Electroretinogram’,” Investigative Ophthalmology & Visual Science 54, no. 1 (2013): 898–908. 10.1167/iovs.12-10971 PMC3880354 23221069

[11] A. G. Robson , A. R. Webster , M. Michaelides , et al., “‘Cone Dystrophy With Supernormal Rod Electroretinogram’: A Comprehensive Genotype/Phenotype Study Including Fundus Autofluorescence and Extensive Electrophysiology,” Retina 30, no. 1 (2010): 51–62. 10.1097/IAE.0b013e3181bfe24e 19952985

[12] K. R. Alexander and G. A. Fishman , “Supernormal Scotopic ERG in Cone Dystrophy,” British Journal of Ophthalmology 68, no. 2 (1984): 69–78. 10.1136/bjo.68.2.69 PMC1040261 6607068

[13] T. A. C. de Guimaraes , M. Georgiou , A. G. Robson , et al., “KCNV2‐Associated Retinopathy: Genotype–Phenotype Correlations—KCNV2 Study Group Report 3,” British Journal of Ophthalmology 108, no. 8 (2024): 1137–1144. 10.1136/bjo-2023-323640 PMC11287651 37852740

[14] S. Ben Salah , S. Kamei , A. Sénéćhal , et al., “Novel KCNV2 Mutations in Cone Dystrophy With Supernormal Rod Electroretinogram,” American Journal of Ophthalmology 145, no. 6 (2008): 1099–1106. 10.1016/j.ajo.2008.02.004 18400204

[15] M. Georgiou , A. G. Robson , K. Fujinami , et al., “KCNV2‐Associated Retinopathy: Genetics, Electrophysiology, and Clinical Course‐KCNV2 Study Group Report 1,” American Journal of Ophthalmology 225 (2021): 95–107. 10.1016/j.ajo.2020.11.022 PMC8186730 33309813

[16] K. E. Smith , S. E. Wilkie , J. T. Tebbs‐Warner , et al., “Functional Analysis of Missense Mutations in Kv8.2 Causing Cone Dystrophy With Supernormal Rod Electroretinogram,” Journal of Biological Chemistry 287, no. 52 (2012): 43972–43983. 10.1074/jbc.M112.388033 PMC3527980 23115240

[17] P. I. Sergouniotis , G. E. Holder , A. G. Robson , et al., “High‐Resolution Optical Coherence Tomography Imaging in KCNV2 Retinopathy,” British Journal of Ophthalmology 96, no. 2 (2012): 213–217. 10.1136/bjo.2011.203638 21558291

[18] S. Alghadban , A. Bouchareb , R. Hinch , et al., “Electroporation and Genetic Supply of Cas9 Increase the Generation Efficiency of CRISPR/Cas9 Knock‐In Alleles in C57BL/6J Mouse Zygotes,” Scientific Reports 10, no. 1 (2020): 17912. 10.1038/s41598-020-74960-7 PMC7578782 33087834

[19] L. Kinder and M. Lindner , “Expression of Osteopontin in M2 and M4 Intrinsically Photosensitive Retinal Ganglion Cells in the Mouse Retina,” Investigative Ophthalmology & Visual Science 66, no. 2 (2025): 14. 10.1167/iovs.66.2.14 PMC11804889 39908128

[20] E. Carpentiero , S. Hughes , J. Rodgers , et al., “Interaction Between Native and Prosthetic Visual Responses in Optogenetic Visual Restoration,” JCI Insight 10, no. 11 (2025): e189447. 10.1172/jci.insight.190785 PMC12220941 40232851

[21] S. Biswas , E. Carpentiero , N. Xhaferri , et al., “Lipid Nanoparticles Enable mRNA Delivery to Diverse Cell Types of the Inner Retina,” Molecular Therapy: Nucleic Acids 37, no. 3 (2026): 102996. 10.1016/j.omtn.2026.102996 PMC13382181 42519573

[22] J. Schindelin , I. Arganda‐Carreras , E. Frise , et al., “Fiji: An Open‐Source Platform for Biological‐Image Analysis,” Nature Methods 9, no. 7 (2012): 676–682. 10.1038/nmeth.2019 PMC3855844 22743772

[23] H. Wickham , ggplot2: Elegant Graphics for Data Analysis (Springer‐Verlag, 2016).

[24] R Core Team , R: A Language and Environment for Statistical Computing (R Core Team, 2021), https://www.R‐project.org.

[25] M. Lindner , “The ERGtools2 Package: A Toolset for Processing and Analysing Visual Electrophysiology Data,” Documenta Ophthalmologica 150 (2025): 169–175. 10.1007/s10633-025-10017-2 PMC12137496 40220212

[26] C. Hippert , A. B. Graca , A. C. Barber , et al., “Muller Glia Activation in Response to Inherited Retinal Degeneration Is Highly Varied and Disease‐Specific,” PLoS One 10, no. 3 (2015): e0120415. 10.1371/journal.pone.0120415 PMC4368159 25793273

[27] S. Ahuja , P. Ahuja , A. R. Caffé , P. Ekström , M. Abrahamson , and T. van Veen , “rd1 Mouse Retina Shows Imbalance in Cellular Distribution and Levels of TIMP‐1/MMP‐9, TIMP‐2/MMP‐2 and Sulfated Glycosaminoglycans,” Ophthalmic Research 38, no. 3 (2006): 125–136. 10.1159/000090533 16374054

[28] K. D. Kolstad , D. Dalkara , K. Guerin , et al., “Changes in Adeno‐Associated Virus‐Mediated Gene Delivery in Retinal Degeneration,” Human Gene Therapy 21, no. 5 (2010): 571–578. 10.1089/hum.2009.194 PMC3143418 20021232

[29] W. Yu , J. Xu , and M. Li , “NAB Domain Is Essential for the Subunit Assembly of Both Alpha‐Alpha and Alpha‐Beta Complexes of Shaker‐Like Potassium Channels,” Neuron 16, no. 2 (1996): 441–453. 10.1016/s0896-6273(00)80062-8 8789959

[30] S. M. Inamdar , C. K. Lankford , D. Poria , et al., “Differential Impact of Kv8.2 Loss on Rod and Cone Signaling and Degeneration,” Human Molecular Genetics 31, no. 7 (2022): 1035–1050. 10.1093/hmg/ddab301 PMC8976434 34652420

[31] A. R. Mackie and K. L. Byron , “Cardiovascular KCNQ (Kv7) Potassium Channels: Physiological Regulators and New Targets for Therapeutic Intervention,” Molecular Pharmacology 74, no. 5 (2008): 1171–1179. 10.1124/mol.108.049825 18684841

[32] C. J. Jeon , E. Strettoi , and R. H. Masland , “The Major Cell Populations of the Mouse Retina,” Journal of Neuroscience 18, no. 21 (1998): 8936–8946. 10.1523/JNEUROSCI.18-21-08936.1998 PMC6793518 9786999

[33] D. Zobor , S. Kohl , B. Wissinger , E. Zrenner , and H. Jägle , “Rod and Cone Function in Patients With KCNV2 Retinopathy,” PLoS One 7, no. 10 (2012): e46762. 10.1371/journal.pone.0046762 PMC3471896 23077521

[34] N. S. Hart , J. K. Mountford , V. Voigt , et al., “The Role of the Voltage‐Gated Potassium Channel Proteins Kv8.2 and Kv2.1 in Vision and Retinal Disease: Insights From the Study of Mouse Gene Knock‐Out Mutations,” eNeuro 6, no. 1 (2019): ENEURO.0032–ENEU19.2019. 10.1523/ENEURO.0032-19.2019 PMC6393689 30820446

[35] T. Sato , K. Kuniyoshi , T. Hayashi , H. Nishiwaki , K. Mizobuchi , and S. Kusaka , “Clinical Course of Two Siblings With Potassium Voltage‐Gated Channel Modifier Subfamily V Member 2 (KCNV2)‐Associated Retinopathy,” Documenta Ophthalmologica 148, no. 3 (2024): 173–182. 10.1007/s10633-024-09971-0 38630375

[36] C. A. Curcio , K. R. Sloan , R. E. Kalina , and A. E. Hendrickson , “Human Photoreceptor Topography,” Journal of Comparative Neurology 292, no. 4 (1990): 497–523. 10.1002/cne.902920402 2324310

[37] R. A. Masri , U. Greferath , E. L. Fletcher , et al., “Immunohistochemistry and Spatial Density of Muller Cells in the Human Fovea,” Investigative Ophthalmology & Visual Science 66, no. 2 (2025): 46. 10.1167/iovs.66.2.46 PMC11838121 39964323

[38] M. Georgiou , K. Fujinami , A. Vincent , et al., “KCNV2‐Associated Retinopathy: Detailed Retinal Phenotype and Structural Endpoints‐KCNV2 Study Group Report 2,” American Journal of Ophthalmology 230 (2021): 1–11. 10.1016/j.ajo.2021.03.004 PMC8710866 33737031

