# Supplementary material for: Kcnv2 E151X Mouse Captures Hallmarks of KCNV2‐Associated Retinal Dystrophy
Source: Clin Exp Ophthalmol. 2026 Sep 1;54(8):1041–53. doi: 10.1111/ceo.70163 (PMC13741982; doi:10.1111/ceo.70163)
Supplement: Supplementary file 1 [file CEO-54-1041-s001.docx]

# Supplemental figures

Figure S1. Representative retinal sections immunolabeled for Kv8.2 in WT (A) and Kcnv2^E151X/E151X^ mice (B). Note cryosections shown here are from mice different to those shown in Figure 2. Primary antibody used here is the same as in Figure 2, only a different, red fluorescent, secondary antibody was used. Weak residual KCNV2 immunostaining was occasionally detectable in some Kcnv2^E151X/E151X^ retinas. (Scale bar = 10 µm;)

Figure S2. Raw Western blot for Kcnv2 in retinal lysates from three genotypes. Representative unprocessed Western Blot image showing Kcnv2 protein (green) and GAPDH (red) expression in retinal lysates from WT, heterozygous and E151X mice.

Figure S3. Cone arrestin immunostaining in retinal wholemounts. (A) Representative retinal wholemount images from 36 weeks old E151X/E151X mice (top) and age-matched WT/E151X mice (bottom) stained for cone arrestin. Scale bar = 10 µm. (B) Quantification of cone density in retinal wholemounts. Three mice were analyzed per genotype (n = 3) with individual data points representing measurements obtained from three independent retinal regions per mouse, excluding the far periphery. Data are shown as mean ± SEM. Statistical significance was determined using an unpaired two-tailed Student's t-test.

# References
